# Limonene, a Monoterpene, Mitigates Rotenone-Induced Dopaminergic Neurodegeneration by Modulating Neuroinflammation, Hippo Signaling and Apoptosis in Rats

**DOI:** 10.3390/ijms24065222

**Published:** 2023-03-09

**Authors:** Lujain Bader Eddin, Sheikh Azimullah, Niraj Kumar Jha, Mohamed Fizur Nagoor Meeran, Rami Beiram, Shreesh Ojha

**Affiliations:** 1 Department of Pharmacology and Therapeutics, College of Medicine and Health Sciences, United Arab Emirates University, Al Ain P.O. Box 15551, United Arab Emirates; 2 School of Bioengineering and Biosciences, Lovely Professional University, Phagwara 144411, Punjab, India; 3 Zayed Bin Sultan Center for Health Sciences, United Arab Emirates University, Al Ain P.O. Box 15551, United Arab Emirates

**Keywords:** rotenone, limonene, monoterpenes, neurodegeneration, dopaminergic neurons, neuroinflammation, Parkinson’s disease

## Abstract

Rotenone (ROT) is a naturally derived pesticide and a well-known environmental neurotoxin associated with induction of Parkinson’s disease (PD). Limonene (LMN), a naturally occurring monoterpene, is found ubiquitously in citrus fruits and peels. There is enormous interest in finding novel therapeutic agents that can cure or halt the progressive degeneration in PD; therefore, the main aim of this study is to investigate the potential neuroprotective effects of LMN employing a rodent model of PD measuring parameters of oxidative stress, neuro-inflammation, and apoptosis to elucidate the underlying mechanisms. PD in experimental rats was induced by intraperitoneal injection of ROT (2.5 mg/kg) five days a week for a total of 28 days. The rats were treated with LMN (50 mg/kg, orally) along with intraperitoneal injection of ROT (2.5 mg/kg) for the same duration as in ROT-administered rats. ROT injections induced a significant loss of dopaminergic (DA) neurons in the substantia nigra pars compacta (SNpc) and DA striatal fibers following activation of glial cells (astrocytes and microglia). ROT treatment enhanced oxidative stress, altered NF-κB/MAPK signaling and motor dysfunction, and enhanced the levels/expressions of inflammatory mediators and proinflammatory cytokines in the brain. There was a concomitant mitochondrial dysfunction followed by the activation of the Hippo signaling and intrinsic pathway of apoptosis as well as altered mTOR signaling in the brain of ROT-injected rats. Oral treatment with LMN corrected the majority of the biochemical, pathological, and molecular parameters altered following ROT injections. Our study findings demonstrate the efficacy of LMN in providing protection against ROT-induced neurodegeneration.

## 1. Introduction

Parkinson’s disease (PD) is one of the prevalent slow progressing disorders, pathologically featured by the degeneration of dopamine-producing neurons in substantia nigra pars compacta (SNpc), which culminates in disabling motor dysfunctions, such as bradykinesia, muscular rigidity, resting tremor, and gait impairment [1,2]. Compelling experimental evidence implicates oxidative damage, inflammation, mitochondrial dysfunction and apoptosis as major factors in the induction and progression of PD [3]. 

Neuroinflammation is an intrinsic player in PD progression that apparently reflects the role of microglia in PD [4]. Activated microglia produces proinflammatory cytokines causing neurotoxicity as well as chemokines that can recruit leukocytes to the central nervous system, exacerbating inflammatory response [5]. In recent years, substantial evidence on mitochondrial dysfunction and apoptosis in the etiopathogenesis of PD has been demonstrated [6]. Various studies demonstrate that impaired mitochondrial bioenergetics and dysfunction, especially perturbations in electron transport chain (ETC) complex-I, play an important role in the pathogenesis of PD [7].

Available evidence revealed a strong correlation between the levels of α-synuclein and disease severity. Malfunction of the proteolytic machineries responsible for α-synuclein degradation may result in the aggregation and accumulation of the protein, which characterize the disease [8]. Consequently, accumulated α-synuclein results in oxidative stress and neuro-inflammation which are the two substantial pathological mechanisms in PD [9]. Overexpressed α-synuclein, especially in its mutant form, is associated with an increased rate of neuronal cell death [10]. These pathological events finally result in dopamine depletion following the degeneration of dopaminergic neurons and the emergence of motor symptoms in PD. Hence, oxidative stress, neuroinflammation, and apoptotic cell death of dopaminergic neurons emerged as vital targets for therapeutic intervention in PD [11]. Currently, there are no available pharmacological agents to completely cure or delay or halt the progression of PD. Levodopa is still considered the most effective symptomatic reliever in controlling the severity of symptoms associated with PD. Therefore, there is a need for developing agents which can target complex interlinked cascades involved in PD [12]. 

Rotenone (ROT), a naturally occurring pesticide, is widely used for the induction of dopaminergic neurodegeneration in experimental animals. It is a specific inhibitor of mitochondrial complex I of the respiratory chain, producing free-radical-mediated oxidative stress [13]. The model of ROT-induced dopaminergic neurodegeneration that represents features of PD is more advantageous than other chemical-based experimental models of PD as it resembles the neurological and behavioral changes of PD reflecting the selective degeneration of dopaminergic neurons [14]. ROT-injected animals evidently mimic the pathological features of PD in patients, such as the progressive loss of dopaminergic neurodegeneration, α-synuclein aggregation in dopaminergic neurons, Lewy body formation, oxidative stress, mitochondrial dysfunction, microglial activation, and neuroinflammation [15]. Therefore, exposing rats to ROT is a relevant and interesting model that has gained popularity in studying PD pathogenesis and in pharmacological evaluations of agents [13]. 

Plant-derived bioactive agents, commonly known as phytochemicals, that are used either as nutraceuticals or dietary supplements or pharmaceuticals, have caught the attention of most recent research due to their high therapeutic potential and low toxicity. Phytochemicals appear to have an inevitable role in the prevention and treatment of chronic diseases, including neurodegeneration. Among plant-derived bioactive compounds, the triterpenoid class of compounds has received enormous attention in recent times. One of the popular triterpenoid compounds, limonene (LMN) has been found to be effective against experimental models of numerous diseases, including neurological disorders such as Alzheimer’s disease [16], stroke [17], and cerebral ischemia [18]. LMN exhibits a variety of biological properties, such as antioxidant, anti-inflammatory, anticancer, gastroprotective, neuroprotective [19] properties, and induction of autophagy [20], along with negligible toxicity [21,22]. LMN has been reported to be absorbed considerably and undergoes rapid biotransformation to active metabolites (perillic acid, dihydroperillic acid, and limonene-1,2-diol) in both humans and animal models [23,24,25]. LMN is excreted in the urine within 48 h in humans and the half-life ranges from 12 to 24 h [25]. Considering the neuroprotective potential and favorable pharmacokinetic properties and druggability of LMN, it is imperative to investigate the effects of LMN on dopaminergic neurodegeneration, a characteristic of PD. Thus, this present study aimed to evaluate the therapeutic potential of LMN in ROT-induced PD in rats. Further, the underlying mechanism was determined by evaluating markers of oxidative stress, neuroinflammation, and apoptotic signaling along with immunostaining of dopaminergic neurons. 

## 2. Results

### 2.1. Behavioral Assessment of the Impact of Limonene on ROT-Induced Neurodegeneration

**Figure 1:**
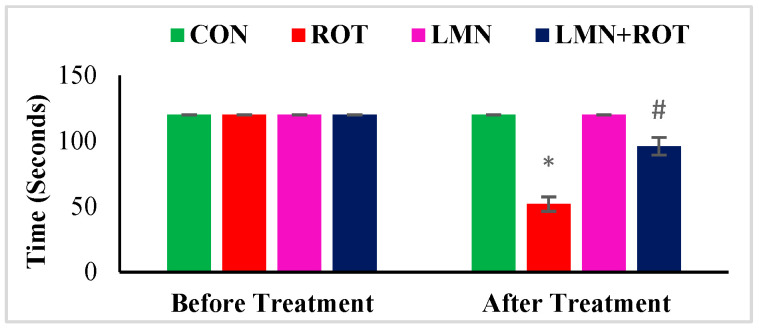
Rotarod test performance of rats. The values are presented as mean ± SEM. * *p* < 0.05 CON vs. ROT; ^#^ *p* < 0.05 ROT vs. LMN + ROT (one-way ANOVA followed by DMRT). CON: normal control, ROT: rotenone, LMN: limonene, LMN + ROT: limonene and rotenone treatment.

To evaluate the effect of LMN on ROT-induced dyskinesia, a rotarod test was performed to measure rats’ ability to maintain themselves on the rotating rod. Behavioral test results showed a gradual decrease in balance level and muscle strength in ROT-injected rats (Figure 1). Rats injected with ROT displayed a significant (*p* < 0.05) decline in rotarod performance compared to normal control rats. LMN treatment to the rats injected with ROT showed improved ability to balance as observed in the rotarod test (Figure 1). It decreased the latency time to fall, where it showed a significant (*p* < 0.05) increase in retention time on the rotarod as compared with ROT-injected rats. LMN treatment did not affect the performance of rats compared to the normal control group (Figure 1).

### 2.2. Limonene Preserves Dopaminergic Neurons against ROT-Induced Neurodegeneration 

**Figure 2:**
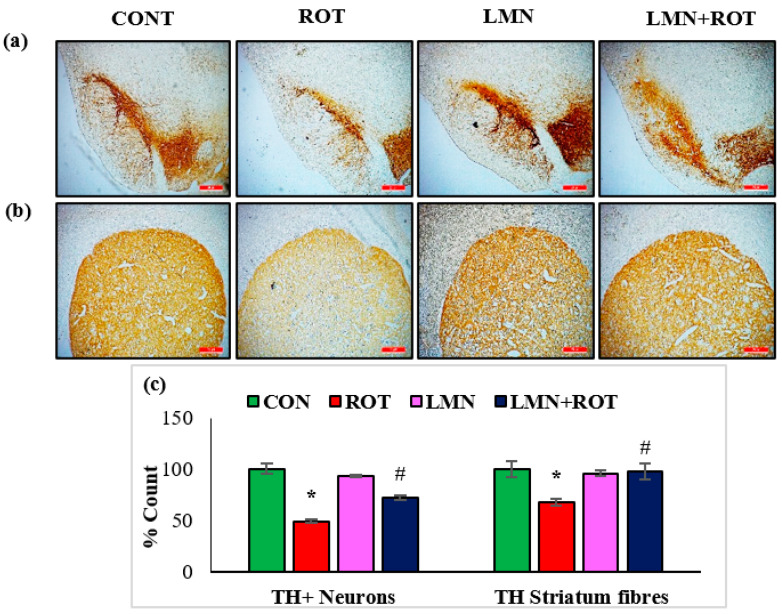
TH-ir neurons and TH-ir fibers in the substantia nigra (SN) and striatum are presented, respectively (**a**,**b**) (scale bar is 20 µm). (**c**) Quantification of the TH-ir neurons in SNc and the density of TH-ir fibers is also shown. Each group contains three rats, and the data are represented as percent mean ± SEM. * *p* < 0.05 CON vs. ROT; ^#^ *p* < 0.05 ROT vs. LMN + ROT (one-way ANOVA followed by DMRT). CON: normal control, ROT: rotenone, LMN: limonene, LMN + ROT: limonene and rotenone treatment.

To investigate the effect of LMN on ROT-induced alterations in the nigrostriatal system in rats, the loss of dopaminergic neurons was detected by staining the TH-ir neurons and their corresponding fibers in the striatum. Immunohistochemistry of TH assessed the presence of TH-ir dopaminergic neurons in the SN and TH-ir dopaminergic striatal fibers. ROT-injected rats showed a significant (*p* < 0.05) reduced number of TH-positive cells in the SN and a reduced density of striatal TH-ir fibers (Figure 2). LMN treatment to ROT-injected rats decreased the loss of TH-positive neurons, evidenced by the increased number of dopaminergic neurons in SN and TH-ir fibers in striatum. In the normal-control- and LMN-treated groups, the SN contained large numbers of TH-immunopositive neurons along with dopaminergic content in the striatum. This suggests that LMN treatment can reduce the loss of dopaminergic neurons caused by ROT (Figure 2).

### 2.3. Effect of Limonene on Brain-Derived Neurotrophic Factor (BDNF) and α-Synuclein Expression in the Striatum of Rats

**Figure 3:**
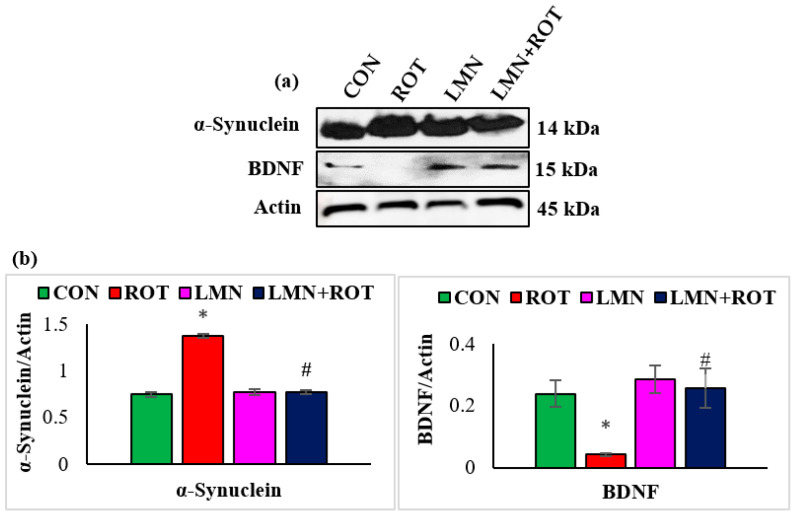
Representative images of Western blots and densitometric analysis for BDNF and α-synuclein expressions in the striatal tissues (**a**,**b**). Immunoblotting was performed in duplicates and the data are expressed as the mean ± SEM. * *p* < 0.05 CON vs. ROT; ^#^ *p* < 0.05 ROT vs. LMN + ROT (one-way ANOVA followed by DMRT). CON: normal control, ROT: rotenone, LMN: limonene, LMN + ROT: limonene and rotenone treatment.

Inefficient neuronal supply of BDNF can lead to PD due to the defects in synaptic plasticity associated with BDNF loss. To investigate the effects of LMN on BDNF, the expressions of BDNF in striatum of different experimental groups were studied (Figure 3). A significant decrease (*p* < 0.05) in the BDNF protein expression was observed, whereas LMN treatment was observed to be associated with increased expression of BDNF, which reflects a prevention of the loss of BDNF. To further confirm that LMN treatment protects neurons, we measured the level of α-synuclein, which accounts for deleterious effects following abnormal accumulation in dopaminergic neurons. Western blot analysis reveals that ROT injections induced a significant increase (*p* < 0.05) in expression of α-synuclein in the striatum when compared to the normal control rats (Figure 3). However, LMN treatment reduced the expression of α-synuclein that could be indicative of a reduced accumulation of α-synuclein in comparison with the rats that received only ROT (Figure 3). 

### 2.4. Limonene Attenuates Lipid Peroxidation and Enhances the Activities/Concentrations of Enzymatic and Non-Enzymatic Antioxidant Status in the Midbrain of ROT-Induced Neurodegeneration

**Figure 4:**
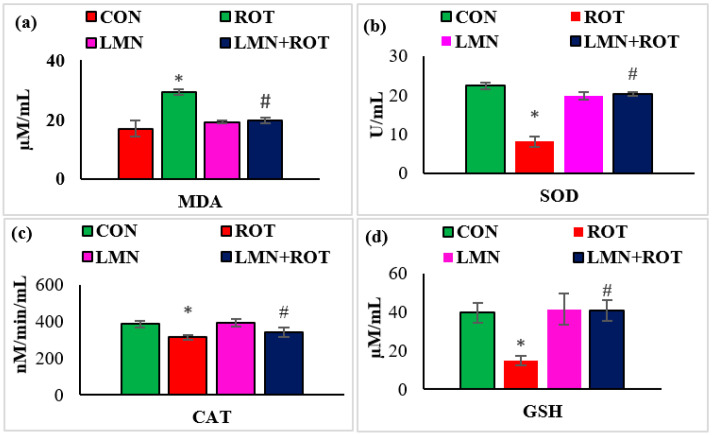
Effect of limonene on the levels of MDA, SOD, catalase (CAT), and GSH in the midbrain of rats (**a**–**d**). The values are presented as mean ± SEM (n = 6–8); * *p* < 0.05 CON vs. ROT; ^#^ *p* < 0.05 ROT vs. LMN + ROT (one-way ANOVA followed by DMRT). CON: normal control, ROT: rotenone, LMN: limonene, LMN + ROT: limonene and rotenone treatment.

The antioxidant effect of LMN was evaluated by assessing the activity of antioxidant enzymes and the extent of peroxidized compounds formed in the midbrain. The MDA levels were considerably (*p* < 0.05) increased whereas the activities of SOD, catalase, and concentrations of GSH were remarkably (*p* < 0.05) reduced in ROT-injected rats compared to normal control rats (Figure 4). However, LMN treatment significantly (*p* < 0.05) reduced the MDA levels and significantly (*p* < 0.05) improved the activities/concentrations of SOD, catalase, and GSH in rats that received ROT compared to the rats only injected with ROT (Figure 4).

### 2.5. Limonene Attenuates Proinflammatory Cytokines in the Midbrain of ROT-Induced Neurodegeneration 

**Figure 5:**
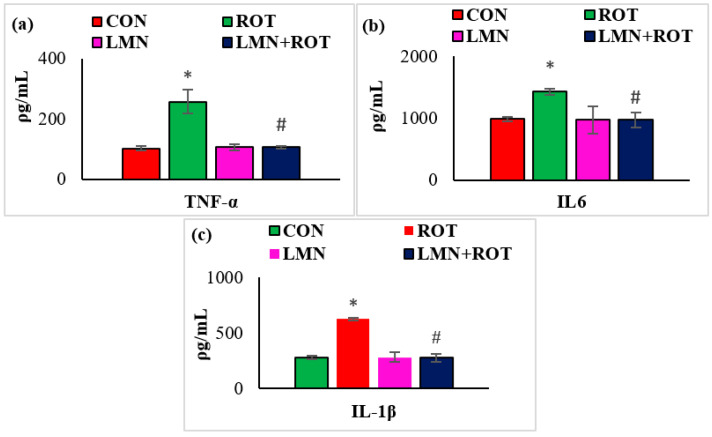
Effect of limonene on the concentration of proinflammatory cytokines (TNF-α, IL-6, and IL-1β) in the midbrain of rats (**a**–**c**). The values are presented as mean ± SEM (n = 6–7); * *p* < 0.05 CON vs. ROT; ^#^ *p* < 0.05 ROT vs. LMN + ROT (one-way ANOVA followed by DMRT). CON: normal control, ROT: rotenone, LMN: limonene, LMN + ROT: limonene and rotenone treatment.

Neuroinflammation is considered one of the key pathogenic events that plays a role in the progression of neurodegeneration. Therefore, we investigated the effect of LMN on ROT-induced neuroinflammation by measuring the levels of the proinflammatory cytokines (TNF-α, IL-1β, and IL-6) in the midbrain of rats. ROT injection to the rats caused a considerable (*p* < 0.05) rise in the levels of TNF-α, IL-1β, and IL-6 in the midbrain compared to normal control rats (Figure 5), whereas treatment with LMN produced a significant (*p* < 0.05) reduction in the levels of these proinflammatory cytokines in the midbrain compared to only ROT-injected rats (Figure 5). 

### 2.6. Limonene Attenuates Activation of Microglia and Astrocytes in ROT-Induced Neurodegeneration

**Figure 6:**
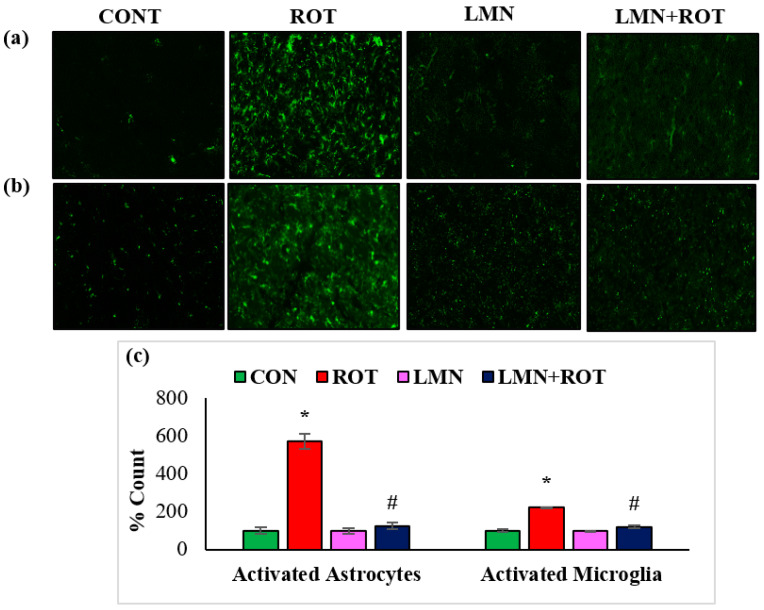
The activation of GFAP and Iba-1 in striatum was explored by immunofluorescence staining of striatum. A remarkable expression of activated astrocytes (GFAP-positive) (**a**), and microglia (Iba-1-positive) (**b**), was indicated in the fluorescent images taken from ROT-injected rats when compared to the control rats. (**c**), The quantitative analysis of the number of activated astrocytes and microglia is shown. Each group consists of three rats and the values are presented as mean ± SEM; * *p* < 0.05 CON vs. ROT; ^#^ *p* < 0.05 ROT vs. LMN + ROT (one-way ANOVA followed by DMRT). CON: normal control, ROT: rotenone, LMN: limonene, LMN + ROT: limonene and rotenone treatment.

Activation of GFAP and Iba-1 are considered as markers of ROS production and inflammatory process. Immunofluorescence staining of GFAP and Iba-1 in the striatum shows the high number of activated GFAP-positive astrocytes (Figure 6a) and Iba-1-positive microglia (Figure 6b) in ROT-injected rats compared to normal control rats. This is conjectured by the increased number as well as size of astrocytes and microglia. However, LMN treatment to ROT-administered rats showed reduced activation of astrocytes and microglia when compared to ROT-injected rats. The quantification of activated astrocytes and microglia are presented in Figure 2. Consistently, there is activation of more astrocytes and microglia in ROT-injected rats in comparison with the control rats, while LMN treatment exerted a reduction in the number of activated astrocytes and microglia when compared with rats injected with ROT only. Normal-control-treated rats and those treated with LMN only did not display notable expression of astrocytes and microglia, which is suggestive of no adverse effects on astrocytes and microglia with the studied doses. 

### 2.7. Limonene Treatment Inhibits Expression of Inflammatory Mediators and NF-κB/IκB Activation in the Striatum of ROT-Induced Neurodegeneration 

**Figure 7:**
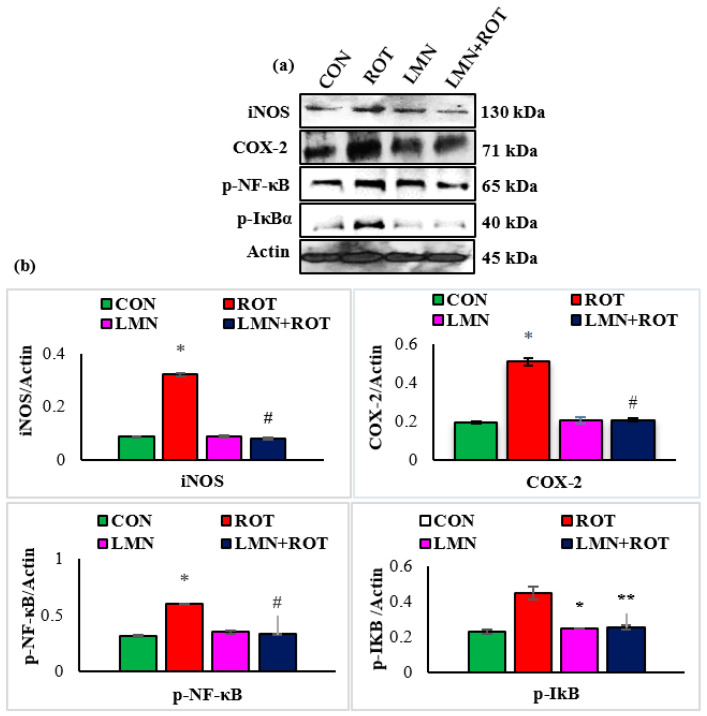
Immunoblotting analysis and quantification of iNOS, COX-2, and p-NF-κB in the striatal tissues of rats (**a**,**b**). Immunoblotting was performed in duplicate and the results are presented as mean ± SEM. * *p* < 0.05 CON vs. ROT; ^#^ *p* < 0.05 ROT vs. LMN + ROT (one-way ANOVA followed by DMRT). CON: normal control, ROT: rotenone, LMN: limonene, LMN + ROT: limonene and rotenone treatment.

To further characterize the inhibitory effect of LMN on mediators of inflammation, we investigated the activation of NF-κB via Western blotting. ROT-injected rats showed a significant (*p* < 0.05) rise in the protein expressions of iNOS, COX-2, p-NF-κB, and p-IκB in the striatum of rats (Figure 7). However, LMN treatment to ROT-injected rats produced a significant (*p* < 0.05) reduction in the expressions of iNOS, COX-2, p-NF-κB, and p-IκB in the striatum. Rats injected with LMN alone did not exhibit any noticeable alteration in the expressions of all these proteins in the striatum (Figure 7).

### 2.8. Limonene Treatment Reduces Phosphorylation of MAPK Signaling Proteins in the Striatum of ROT-Induced Neurodegeneration

**Figure 8:**
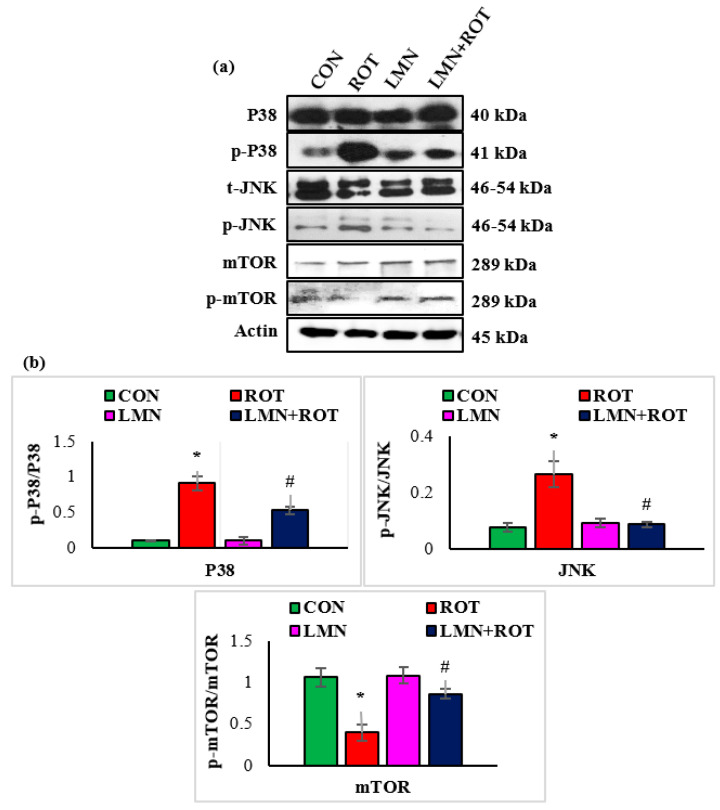
Effect of limonene on the protein expressions of P38, JNK, m-TOR, and p-m-TOR densitometric analysis in the striatal tissues (**a**,**b**). Immunoblotting was performed in duplicate and the results are presented as mean ± SEM. * *p* < 0.05 CON vs. ROT; ^#^ *p* < 0.05 ROT vs. LMN + ROT (one-way ANOVA followed by DMRT). CON: normal control, ROT: rotenone, LMN: limonene, LMN + ROT: limonene and rotenone treatment.

To confirm whether LMN regulates the role of the P38 MAPK and mTOR signaling pathways in PD induction, we further investigated p-JNK, p-p38, and p-mTOR activation in different experimental groups. Phosphorylation of JNK and P38 was significantly (*p* < 0.05) increased in ROT-injected rats in comparison with normal control rats (Figure 8), whereas LMN treatment to ROT-injected rats produced a significant (*p* < 0.05) reduction in the phosphorylation of MAPK proteins when compared to only ROT injected rats. Phosphorylated mTOR was downregulated in ROT-injected rats, whereas LMN treatment for ROT-injected rats restored normal expression. Both normal-control-, and LMN-alone-treated rats had no notable change in the expression of these phosphorylated MAPK signaling proteins (Figure 8).

### 2.9. Limonene Inhibits ROT-Induced Mitochondrial Complex-I Inhibition in the Striatum

**Figure 9:**
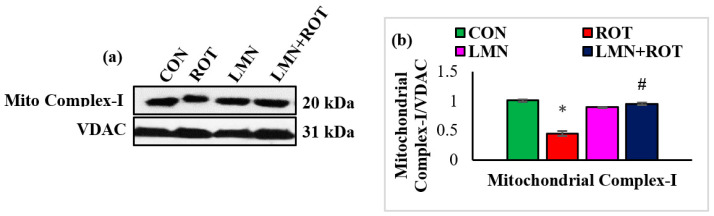
Western immunoblotting and densitometric analysis on the protein expression of mitochondrial complex-I in the striatal tissues of rats (**a**,**b**). Immunoblotting was performed in duplicate and the values are presented as mean ± SEM. * *p* < 0.05 CON vs. ROT; ^#^ *p* < 0.05 ROT vs. LMN + ROT (one-way ANOVA followed by DMRT). CON: normal control, ROT: rotenone, LMN: limonene, LMN + ROT: limonene and rotenone treatment.

ROT is well known as a complex-I inhibitor. Therefore, we evaluated complex-I to ascertain the effect of LMN in ROT-induced PD. The expression of mitochondrial complex-I was significantly decreased (*p* < 0.05) in ROT-injected rats compared to normal control rats, whereas LMN treatment produced a significant (*p* < 0.05) rise in the expressions of mitochondrial complex-I compared to only ROT injected rats (Figure 9).

### 2.10. Limonene Treatment Attenuates Apoptosis and Hippo Signaling in ROT-Injected Rats

**Figure 10:**
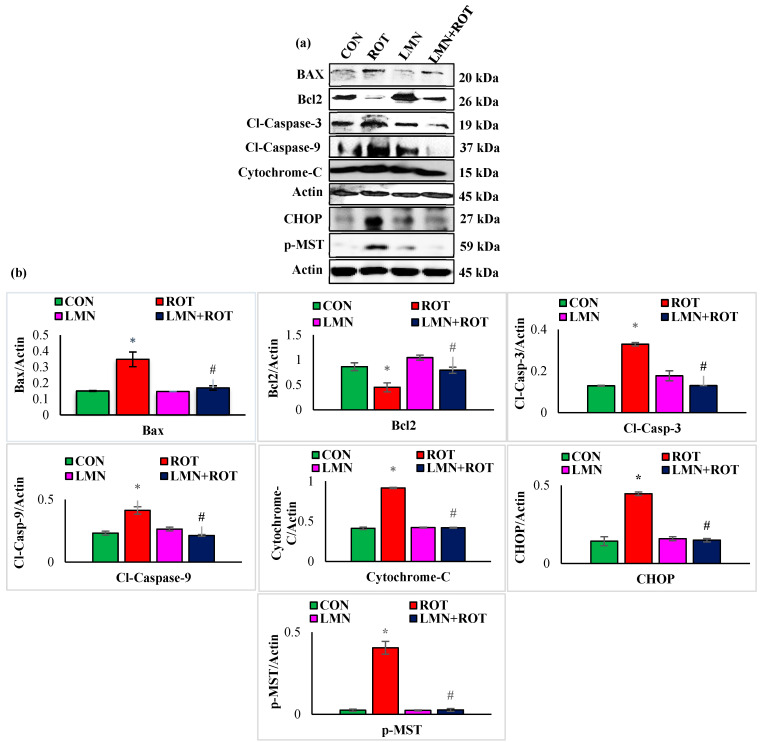
Immunoblotting and densitometric analysis of Bcl2. Antiapoptotic protein and pro-apoptotic proteins, including Bax, cytochrome-C, cleaved caspase-3, cleaved caspase-9, CHOP, p-MST were evaluated in the striatum (**a**,**b**). Immunoblotting was performed in duplicate and the data are presented as mean ± SEM. * *p* < 0.05 CON vs. ROT; ^#^ *p* < 0.05 ROT vs. LMN + ROT (one-way ANOVA followed by DMRT). CON: normal control, ROT: rotenone, LMN: limonene, LMN + ROT: limonene and rotenone treatment.

The intrinsic pathway of apoptosis is initiated by proteolytic activation of the initiator caspase-9 followed by caspase-3 cleavage and activation. We studied the expression of apoptotic proteins and the change induced following LMN treatment. ROT injections to rats caused a significant (*p* < 0.05) rise in the protein expressions of Bax, cleaved caspase-3, cleaved caspase-9, cytochrome-C, CHOP, and p-MST1 compared to normal control rats (Figure 10), whereas treatment with LMN showed a significant (*p* < 0.05) decrease in the expression of these apoptotic and Hippo signaling proteins compared to only ROT injected rats. In contrast, the antiapoptotic protein Bcl2 was decreased in ROT-injected rats, whereas it was increased following LMN treatment. Expressions of these proteins remained unaffected in normal-control- and LMN-alone-treated rats (Figure 10). 

## 3. Discussion

**Figure 11:**
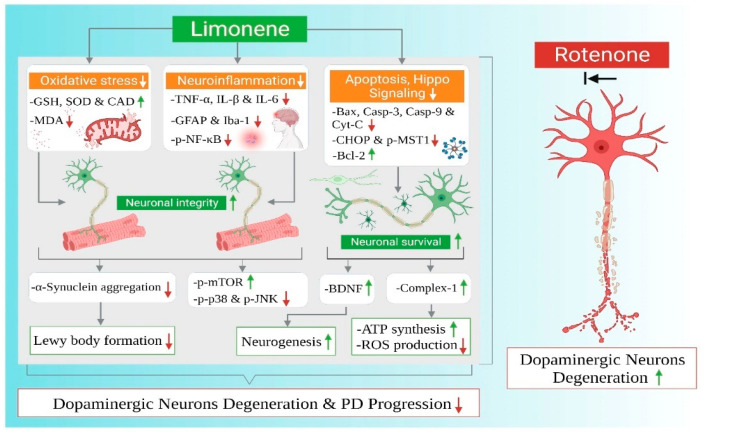
Summarized mechanisms of neuroprotective effect of limonene in experimental model of Parkinson’s disease.

To our knowledge, this current study is the first report on the neuroprotective role of LMN in ROT-induced PD in rats. The neuroprotective effects and mechanisms observed in the present study have been summarized in Figure 11. PD is characterized by a functional impairment of voluntary movements which leads to slowness in fine motor function, including dyskinesia, hypokinesia, or akinesia [26]. PD patients frequently reported difficulties in hand dexterity movements [27]. In our study, we observed reduced time spent on the rotarod for rats injected with ROT, which is consistent with previous reports which concluded that ROT-administered rats remained on the rotating rod for less time compared with pre-ROT injections [28]. However, pretreatment with LMN significantly attenuated ROT-induced locomotor deficits; a sign of the protective ability of LMN against ROT-induced neurodegenerative changes.

ROT as a potent inhibitor of complex-1 of the mitochondrial electron transport chain leads to a syndrome that replicates both the neuropathological and behavioral symptoms of PD. Administration of ROT in vivo has been shown to induce oxidative stress, neuroinflammation, and apoptosis subsequent to both the loss of tyrosine hydroxylase and the formation of fibrillary cytoplasmic inclusions containing α-synuclein. It also induces motor deficits such as bradykinesia and gait instability [27]. ROT has been used for remodeling PD in research since its ability to reproduce the hallmarks of PD was reported [29]. 

Tyrosine hydroxylase is the rate-limiting enzyme of catecholamine biosynthesis converting tyrosine to DOPA. PD affects specifically TH-containing catecholamine neurons. The most prominent neurodegeneration in PD patients is observed in the nigrostriatal DA neurons, which contain abundant TH. Therefore, TH has been speculated to have an essential role in the pathophysiology of PD [30]. In the current study, LMN attenuated the dopaminergic neurodegeneration induced by ROT administration by preventing oxidative stress, reinstating mitochondrial complex-I activity, and maintaining TH-ir neurons in SN.

BDNF as a neurotrophin mediates neurogenesis to maintain neuronal integrity. BDNF signaling through tropomyosin-related kinase B receptor (TrkB) is reported to protect nigrostriatal dopaminergic neurons in aged brains [31]. Imbalanced levels of BDNF can affect the motor and cognitive performance in parkinsonian patients. Deteriorated parkinsonian symptoms are correlated with low BDNF levels in the SN and caudal-putamen nuclei of PD patients [32]. Our study indicated that ROT caused a significant decrease in BDNF expressions, whereas LMN treatment reversed the effect of ROT and enhanced BDNF expressions. 

A critical event in PD that has been supported by strong evidence is α-synuclein accumulation mediating the pathogenesis of PD and leading to other mechanisms representing the cornerstone in PD pathology [33]. α-synuclein aberrant soluble oligomeric conformations, termed protofibrils, are known to mediate disruption of cellular homeostasis and neuronal death. Our data indicate that LMN co-treatment reduced the content of α-synuclein in comparison with ROT only injected rats, suggesting that LMN mediated the inhibition of α-synuclein aggregations. 

Mitochondrial complex-I is a rate-limiting enzyme complex involved in oxidative phosphorylation and ATP synthesis for maintaining the mitochondrial bioenergetics [34]. Intoxication of mitochondrial complex-I reproduces the motor symptoms of PD in experimental models [35]. ROT has a high affinity to inhibit electron transport chain-1 (ETC-I). Persistent inhibition of ETC-I leads to a leakage of electrons, which combine with O_2_ results in excessive ROS production [36]. LMN treatment restored the activity of complex-I and maintained its function. 

Dopaminergic neurons in PD exist in a state of constant oxidative stress, partly due to the free radical generation [37]. Free-radical-induced lipid peroxidation and oxidative stress play a crucial role in PD pathogenesis [38]. The loss of antioxidant defenses leads to a buildup of ROS which is associated with deleterious effects on dopaminergic neurons in PD [39]. ROT-administered rats showed a decline in antioxidant defense by decreasing the activities/concentrations of SOD, catalase, and GSH in the midbrain. Decreased activities/concentrations of SOD, catalase, and GSH in ROT-injected rats resulted from the inactivation of these enzymatic and non-enzymatic antioxidants by H_2_O_2_. Previous studies have revealed that LMN is a potent scavenger of toxic free radicals, such as hydrogen peroxide (H_2_O_2_), superoxide, and hydroxyl free radicals, in vitro [40,41]. However, LMN treatment to ROT-injected rats showed near normalized activities/concentrations of SOD, catalase, and GSH ascribed to its potent antioxidant activity. Consistent with previous reports, our study demonstrated that the neuroprotective ability of LMN is attributed to its free radical scavenging properties and preserving the enzymatic and non-enzymatic antioxidants. 

Microglial activation and increased reactive astrocytes are aggravated by the mutated forms of α-synuclein aggregates that act as chemoattractants of microglial migration toward damaged neurons [42]. Activated microglia followed by IκB phosphorylation and its proteolytic degradation trigger NF-κB nuclear activation and translocation which upregulate the inflammatory mediators iNOS and COX-2 and release enormous amounts of proinflammatory cytokines, such as TNF-α, IL-6, and IL-1β, which exacerbate neurodegeneration [43]. Our results showed that ROT-injected rats displayed sustained activation of microglia cells in parallel with an accumulation of inflammatory mediators and cytokines in the midbrain. These changes were remarkably attenuated in rats treated with LMN. The ability of LMN in limiting neuroinflammation is supported by previous reports where a reduction in the expression of IL-1β and TNF-α in the hippocampus of mice exposed to stress following maternal separation has been demonstrated [44]. 

MAPK family proteins are well known to play a role in regulating the release of proinflammatory cytokine gene expressions. P38 MAPK has a crucial role in PD progression through aggravating neuroinflammation by enhancing proinflammatory cytokines through microglial activation [45]. ROT-induced PD results in P38 MAPK upregulation, which plays a primary role in inducing neuroinflammation and apoptosis [46], where it controls NF-κB and the downstream cytokines [47]. Several reports showed that JNK phosphorylation with subsequent phosphorylation of c-Jun increased the production of proinflammatory cytokines [48]. In ROT-induced neurotoxicity in rats, MAPK signaling proteins were activated which were obviously reversed by LMN revealing its anti-inflammatory effect. 

Accumulation of aberrant misfolded proteins within the dopaminergic neurons leads to cytotoxic oxidative stress which activates cell death in PD. Apoptosis is responsible for neuronal loss in PD which is evidenced by the elevated activity of pro-apoptotic proteins in the postmortem brain tissue of PD patients [49]. Mitochondria plays a key role in mediating apoptotic cell death. Bax induces the permeabilization of the outer mitochondrial membrane, causing the release of cytochrome-C from the mitochondrial intermembranous space. This eventually leads to the cleavage of caspase-9, activation of caspase-3, and apoptosis induction [49]. Concomitantly, there has been a rise in the expression of Bax, cleaved caspase-3 and cytochrome-C, with declined Bcl-2 expression in ROT-injected rats. LMN treatment showed an attenuation of apoptosis as reflected by decreased Bax, cleaved caspase-3 and cytochrome-C, and increased Bcl-2 expressions. This demonstrates that LMN possesses the ability to counter ROT-induced neuronal cell death via inhibiting oxidative stress and apoptosis. It has been observed that ROT induces caspase-dependent and independent apoptosis through the suppression of mTOR signaling [50]. The activation of mTOR signaling has been reported to protect the dopaminergic neurons against apoptotic death and degradation [51]. The present study results revealed a similar effect, wherein LMN treatment produced neuroprotective effects by restoration of mTOR expression suppressed in ROT-injected rats. 

The initiation of programmed cell death involves remarkable upregulation of the transcription factor C/EBP-homologous protein (CHOP) that has been implicated in the neuronal death in the context of oxidative and endoplasmic reticulum stress (ER stress). Additionally, the Hippo signaling pathway represented by CHOP and MST has recently had a major emphasis placed on its profound effect in neurodegeneration subsequent to neuroinflammatory changes by potentiating apoptosis, cellular growth inhibition, and tissue degeneration [52]. The Hippo signaling pathway plays a critical role in dopaminergic neuronal loss evidenced by MST1 activation along with activation of oxidative-stress-induced cell death. Activated Hippo/MST1 is coupled with caspase-3 activation that is aligned with a loss of dopaminergic neurons in PD brains [53]. Overexpression of CHOP, an ER stress mediator, has been reported in various models of PD [54] and mediates apoptosis by upregulating the BH3 only family proteins [55]. MST phosphorylation has also been correlated with oxidative-stress-induced apoptosis in cardiomyopathy [56]. Our study findings indicate that ROT triggers the activation of CHOP/p-MST which contribute to neurodegeneration by augmenting the oxidative response and apoptosis. However, the protective effect of LMN against the upregulation of Hippo signaling proteins in the striatum is suggestive of its antiapoptotic property. 

## 4. Materials and Methods

### 4.1. Drugs and Chemicals

Rotenone, dimethyl sulfoxide, miglyol, phosphate-buffered saline, paraformaldehyde, and D-limonene were procured from Sigma Chemicals (St. Louis, MO, USA). The chemicals used in the present study were of analytical grade. 

### 4.2. Experimental Animals

Healthy male albino Wistar rats (260–300 g) were bred in the Animal Research Facility, College of Medicine and Health Sciences (CMHS), United Arab Emirates University (UAEU). The animals were kept in cages and housed in standard laboratory conditions of relative humidity, temperature, and light/dark cycles with unlimited access to commercially available rat chow diet and water ad libitum. A maximum of 5 rats were placed per cage. Experiments were conducted according to the guidelines approved (Approval No. ERA_2017_5500) by the Animal Ethics Committee of the United Arab Emirates University. 

### 4.3. Experimental Protocol and Study Groups

LMN has been evaluated in previous studies and our laboratory studies in the oral dose range of 25 to 100 mg/kg and shown to be effective in mitigating oxidative stress, inflammatory mediators, and favorably modulated cell signaling pathways in experimental models of different diseases [57,58,59]. The oral dose of LMN 50 mg/kg has been found to be effective in experimental models of acute lung injury [59], gastric ulcer [57], ulcerative colitis [58], acute myocardial infarction [60], orofacial pain [61], diabetes [62], and depression [63]. The dose of 50 mg/kg has been chosen for the evaluation in ROT-induced rat model of PD representing dopaminergic neurodegeneration in agreement with the previous studies. For the induction of dopaminergic neurodegeneration in rats mimicking PD in humans, a stock solution of ROT was prepared by dissolving in dimethyl sulfoxide (DMSO). The stock solution was further diluted in mygliol to reach a final concentration of 2.5 mg/mL. The rats were randomly divided into four groups, each containing fifteen. The rats in group I assigned as the normal control (CON) group received vehicle (olive oil) in similar volume to other groups, 5 days a week for a duration of 28 days. The rats in group II assigned as the ROT group were administered ROT (2.5 mg/kg, i.p.), 5 days a week for a period of 28 days. The rats in group III assigned as the LMN group were orally treated with LMN (50 mg/kg) daily, 5 days a week for a period of 28 days. The rats in group IV assigned as the LMN + ROT group were pretreated with LMN (50 mg/kg) and followed by ROT (2.5 mg/kg, i.p.), 5 days a week for a period of 28 days. 

### 4.4. Tissue Collection and Preparation 

At the end of the experimental period, rats were anesthetized using an intraperitoneal injection of pentobarbital sodium (40 mg/kg body weight) and cardiac perfusion was performed by 0.01 M phosphate-buffered solution (PH 7.4) to wash out blood. Midbrain and striatum tissues were snap-frozen in liquid nitrogen and stored at −80 °C until further analysis. Brains used for immunohistochemistry were post-fixed in 4% paraformaldehyde solution for 2 days and immersed in sucrose solution (30%) at 4 °C for 3 days. The sucrose solution was changed daily, and the tissues were sectioned only after noticing they had completely sunk down in the sucrose solution.

### 4.5. Biochemical Studies

Midbrain tissues were homogenized using a tissue homogenizer in potassium chloride buffer mixed appropriately with protease and phosphatase inhibitors acquired from Thermo Fisher Scientific (Rockford, IL, USA). The tissue homogenates were centrifuged at 14,000× *g* for 20 min at 4 °C. Supernatant was collected, stored, and utilized for various biochemical estimations. 

### 4.6. Rotarod Test 

Rotarod test is a well-accepted test for the assessment of neurological disorders in animals and it can be repeatedly applied to each rat to evaluate muscle strength, fore and hind limb motor coordination, and balance of rats [64]. In this test, time was recorded for each rat placed on the rotating rod completing ipsilateral and contralateral rotation. This procedure was performed before the beginning of the treatment, to show no variation among groups and that all rats were healthy. Rats were given three days for training and adaptation. They were allowed on the rotating rod to get familiarized with the test. On the fourth day, the time for which each rat could remain on the rod was recorded. At the end of the treatment, the process was repeated again. The rats were given one day for training and recording took place on the following day. The speed was constant throughout all trials (30 rotations per minute). The data are presented as the mean retaining time on the rotating bar for the rats. 

### 4.7. Immunofluorescence Staining for Glial Fibrillary Acidic Protein (GFAP) and Ionized Calcium-Binding Adapter Molecule 1 (Iba-1)

The GFAP and Iba1 activation was assessed by immunofluorescence staining of the striatum. Floating striatum sections of 25 μm thickness were cut by microtome and kept temporarily in phosphate-buffered solution (PBS) (Sigma Aldrich, St. Louis, MO, USA) with sodium azide to prevent contamination. Selected sections were rinsed twice using PBS and kept for the duration of one hour in blocking solution containing 10% normal goat serum and 0.3% Triton-X 100 (Sigma Aldrich, St. Louis, MO, USA) in PBS. The floating sections were incubated over night at 4 °C with polyclonal rabbit anti-GFAP (1:1000) (Abcam, Waltham, MA, USA) and anti-Iba-1 (1:1000) (Vako Chemicals, Richmond, VA, USA) primary antibodies. On the following day, the primary antibodies were rinsed off and the sections were incubated for one hour with fluorescently conjugated secondary antibody Alexa Fluor^®^ 488 anti-rabbit. Thereafter, the sections were mounted with anti-fade mounting medium Fluoroshield™ (Sigma-Aldrich, St. Louis, MO, USA) and preserved by a cover slip. The slides were viewed and images were collected using fluorescence microscope, EVOS FL (Thermo Fisher Scientific, Waltham, MA, USA).

### 4.8. Assessment of Activated GFAP and Iba-1

Coronal striatal sections were assessed for ROT-induced activation of glial cells. Increased fluorescence intensity and prolonged glial processes indicated the activation of astrocytes and microglia. Different regions with similar areas were analyzed for activated GFAP and Iba-1 and quantified by Image J software (NIH, Bethesda, MD, USA).

### 4.9. Immunohistochemistry of Tyrosine Hydroxylase (TH)

Striatum (0.3 mm of bregma) and substantia nigra (SN) regions (−5 mm from bregma) were sectioned for stereological TH immunostaining analysis. The coronal sections of 25 µm were cut by using a cryostat (Leica, Wetzlar, Germany). The sections were washed with PBS (0.01 M) and blocked with 10% normal goat serum and 0.3% Triton-X 100, for the duration of one hour. Afterwards, rabbit anti-TH primary antibody (1:500) (Millipore, Burlington, MA, USA) was added and kept overnight at 4 °C. On the following day, the sections were rinsed and incubated with biotinylated secondary anti-rabbit antibody (1:1000) for the duration of an hour at room temperature. Visualization of TH immunoreactivity was achieved by incubating sections with avidin–biotin complex (Vector Laboratories Ltd., Burlingame, CA, USA) and 3,3′ diaminobenzidine (DAB). Lastly, the sections stained were mounted on slides using DPX mounting medium and viewed under a light microscope (Olympus, Hamburg, Germany).

### 4.10. Determination of Tyrosine Hydroxylase-Immunoreactive (TH-ir) Dopaminergic Neurons and TH-ir Nerve Fibers Loss

In the visualized sections of SNpc and striatum, the immunoreactive neurons of TH were counted and their percentage was calculated in reference to control. Three unified areas from each section of the striatum were measured for their optical density by Image J software. Counting was carried out by taking into account the stained neuronal nucleus. The optical density of overlapping cortex was measured as background and deducted from the optical density of striatum. Assessment of TH-ir neurons was analyzed by an investigator blind to experimental groups. 

### 4.11. Western Blotting Analysis

Using radioimmunoprecipitation assay buffer (RIPA) (Millipore, Burlington, MA, USA), the striatal tissues were homogenized along with protease and phosphatase inhibitors (Merck Millipore, Burlington, MA, USA) to maximize the protein yield. The homogenized tissue was centrifuged at 12,000 rpm for 20 min at 4 °C to obtain the supernatant. The obtained supernatant was further dissolved in 4× Laemmli sample buffer (Bio-Rad, Hercules, CA, USA) and 2-mercaptoethanol (Sigma Chemicals, St. Louis, MO, USA). The protein content was unified across the samples, separated by polyacrylamide gel electrophoresis, transferred to PVDF membrane (Thermo Fisher Scientific, Rockford, IL, USA), blocked in a blocking solution, and immersed at 4 °C overnight with the following primary antibodies: anti-iNOS (1:2000), anti-phospho-MST (1:500) (Sigma Chemicals, St. Louis, MO, USA), anti-COX-2 (1:1000), anti-cleaved caspase-3 (1:1000), anti-cleaved caspase-9 (1:1000), anti-mTOR (1:1000), anti-phospho-mTOR (1:500), anti-CHOP (1:1000), anti-VDAC (1:2000), anti-phospho-IκBα (1:500), (Cell Signaling Technology, Danvers, MA, USA), anti-α-Syn (1:1000), anti-P38 (1:1000), anti-Cytochrome C (1:2000), anti-mitochondrial complex-I (1:1000), anti-brain-derived neurotropic factor (BDNF) (1:1000), P38 MAPK (1:2000), anti-Bcl-2 (1:500), anti-phospho-P38 MAPK (1:1000), anti-NF-κB (1:1000), anti-phospho-NF-κB (1:500), anti-Bax (1:2500), anti-JNK (1:1000), anti-phospho-JNK (1:5000), anti-β-actin (1:5000) (Santa Cruz Biotechnology, Dallas, TA, USA). The corresponding Horseradish peroxidase-conjugated secondary antibody was added to the membranes and incubated for 1 h at room temperature. The chemiluminescence substrate (Thermo Fisher Scientific, Rockford, IL, USA) was added to facilitate the protein visualization. Densitometric analysis was performed using Image J software.

### 4.12. Protein Estimation

The amount of protein in each sample was determined using commercially available Pierce™ BCA protein assay kit (Thermo Fisher Scientific, Rockford, IL, USA).

### 4.13. Assessment of Enzymatic and Non-Enzymatic Antioxidant Status

Activities of superoxide dismutase (SOD), catalase, and glutathione (GSH) were measured in the midbrain using assay kits (Cayman Chemicals Co., Ann Arbor, MI, USA; Sigma-Aldrich, St. Louis, MO, USA). The activities of SOD, catalase, and GSH were calculated as U/mL, nmol/min/mL, and μM/mL, respectively.

### 4.14. Malondialdehyde (MDA) Assay

Malondialdehyde (MDA) levels were estimated using MDA detection kits (Northwest Life Science, Vancouver, WA, USA). The values are represented as μM/mL.

### 4.15. Assessment of Proinflammatory Cytokines

The levels of proinflammatory cytokines, such as tumor necrosis factor-alpha (TNF-α), interleukin-1β (IL-1β), and interleukin-6 (IL-6), were measured using commercial ELISA kits (BioSource International, Camarillo, CA, USA). The values were represented as pg/mL.

### 4.16. Mitochondrial Extraction from the Striatum

Mitochondrial fraction was isolated from the striatum using a commercially available kit following manufacturer’s protocol (Abcam, Waltham, MA, USA). 

### 4.17. Statistical Analysis

The data are represented as the mean ± standard error of the mean (SEM). The data were statistically analyzed using one-way analysis of variance (ANOVA) followed by Duncan’s multiple range test (DMRT) using SPSS (28.0 version). The criterion of statistical significance was set at *p* ≤ 0.05. 

## 5. Conclusions

The findings of the present study show that LMN prevented behavioral deficits, reduced α-synuclein expression, rescued loss of dopaminergic neurons, mitigated oxidative stress, restored complex-I activity, reduced lipid peroxidation, and downregulated proinflammatory cytokines along with a favorable modulation of apoptotic pathways, including MAPK, mTOR, and Hippo signaling, in ROT-induced PD in rats. Based on these findings, it can be concluded that LMN is capable of protecting dopaminergic neurons and maintaining neuronal integrity attributed to its antioxidant, anti-inflammatory, antiapoptotic, and neurogenesis-enhancing properties. The current study substantiates that LMN may be a promising neuroprotective agent against dopaminergic neurodegeneration, a prominent pathologic feature of PD. Thus, LMN being a molecule of natural origin means that it could be further suggested for nutraceutical as well as pharmaceutical development and subjected to regulatory toxicology and human studies. 

## Data Availability

The data used to support the findings of this study are already incorporated in the result section.

## References

[1] Meireles J., Massano J. (2012). Cognitive impairment and dementia in Parkinson’s disease: Clinical features, diagnosis, and management. Front. Neurol..

[2] Obeso J.A. (2010). Modeling clinical features of neurodegeneration. Nat. Med..

[3] Gibson G.E., Huang H.M. (2004). Mitochondrial enzymes and endoplasmic reticulum calcium stores as targets of oxidative stress in neurodegenerative diseases. J. Bioenerg. Biomembr..

[4] Liddelow S.A., Guttenplan K.A., Clarke L.E., Bennett F.C., Bohlen C.J., Schirmer L., Bennett M.L., Münch A.E., Chung W.S., Peterson T.C. (2017). Neurotoxic reactive astrocytes are induced by activated microglia. Nature.

[5] Ramesh G., MacLean A.G., Philipp M.T. (2013). Cytokines and chemokines at the crossroads of neuroinflammation, neurodegeneration, and neuropathic pain. Mediat. Inflamm..

[6] Luo Y., Hoffer A., Hoffer B., Qi X. (2015). Mitochondria: A therapeutic target for Parkinson’s disease?. Int. J. Mol. Sci..

[7] Zhang X., Du L., Zhang W., Yang Y., Zhou Q., Du G. (2017). Therapeutic effects of baicalein on rotenone-induced Parkinson’s disease through protecting mitochondrial function and biogenesis. Sci. Rep..

[8] Xilouri M., Brekk O.R., Stefanis L. (2016). Autophagy and Alpha-Synuclein: Relevance to Parkinson’s Disease and Related Synucleopathies. Mov. Disord..

[9] He J., Zhu G., Wang G., Zhang F. (2020). Oxidative Stress and Neuroinflammation Potentiate Each Other to Promote Progression of Dopamine Neurodegeneration. Oxid. Med. Cell Longev..

[10] Cookson M.R. (2009). alpha-Synuclein and neuronal cell death. Mol. Neurodegener..

[11] Anusha C., Sumathi T., Joseph L.D. (2017). Protective role of apigenin on rotenone induced rat model of Parkinson’s disease: Suppression of neuroinflammation and oxidative stress mediated apoptosis. Chem. Biol. Interact..

[12] Dauncey M.J. (2013). Genomic and epigenomic insights into nutrition and brain disorders. Nutrients.

[13] Amaral de Brito A.P., Galvão de Melo I., El-Bachá R.S., Guedes R.C.A. (2020). Valeriana officinalis Counteracts Rotenone Effects on Spreading Depression in the Rat Brain in vivo and Protects Against Rotenone Cytotoxicity Toward Rat Glioma C6 Cells in vitro. Front. Neurosci..

[14] Johnson M.E., Bobrovskaya L. (2015). An update on the rotenone models of Parkinson’s disease: Their ability to reproduce the features of clinical disease and model gene–environment interactions. Neurotoxicology.

[15] Zhang N., Dou D., Ran X., Kang T. (2018). Neuroprotective effect of arctigenin against neuroinflammation and oxidative stress induced by rotenone. RSC Adv..

[16] Shin M., Liu Q.F., Choi B., Shin C., Lee B., Yuan C., Song Y.J., Yun H.S., Lee I.S., Koo B.S. (2020). Neuroprotective Effects of Limonene (+) against Aβ42-Induced Neurotoxicity in a Drosophila Model of Alzheimer’s Disease. Biol. Pharm. Bull.

[17] Wang X., Li G., Shen W. (2018). Protective effects of D-Limonene against transient cerebral ischemia in stroke-prone spontaneously hypertensive rats. Exp. Ther. Med..

[18] Sadeghimanesh A., Khalaji-Pirbalouty V., Lorigooini Z., Rafieian-Kopaei M., Torki A., Rabiei Z. (2018). Phytochemical and neuroprotective evaluation of Citrus aurantium essential oil on cerebral ischemia and reperfusion. Bangladesh J. Pharmacol..

[19] Eddin L.B., Jha N.K., Meeran M.F.N., Kesari K.K., Beiram R., Ojha S. (2021). Neuroprotective Potential of Limonene and Limonene Containing Natural Products. Molecules.

[20] Yu X., Lin H., Wang Y., Lv W., Zhang S., Qian Y., Deng X., Feng N., Yu H., Qian B. (2018). d-limonene exhibits antitumor activity by inducing autophagy and apoptosis in lung cancer. Onco Targets.

[21] Kim Y.W., Kim M.J., Chung B.Y., Bang D.Y., Lim S.K., Choi S.M., Lim D.S., Cho M.C., Yoon K., Kim H.S. (2013). Safety evaluation and risk assessment of d-Limonene. J. Toxicol. Env. Health B Crit. Rev..

[22] Almeida A.A.C., Ferreira J.R.O., de Carvalho R.B.F., Rizzo M.D.S., Lopes L.D.S., Dittz D., Castro E.S.J.M., Ferreira P.M.P. (2020). Non-clinical toxicity of (+)-limonene epoxide and its physio-pharmacological properties on neurological disorders. Naunyn Schmiedebergs Arch. Pharm..

[23] Chen H., Chan K.K., Budd T. (1998). Pharmacokinetics of d-limonene in the rat by GC-MS assay. J. Pharm. Biomed Anal..

[24] Igimi H., Nishimura M., Kodama R., Ide H. (1974). Studies on the metabolism of d-limonene (p-mentha-1,8-diene). I. The absorption, distribution and excretion of d-limonene in rats. Xenobiotica.

[25] Vigushin D.M., Poon G.K., Boddy A., English J., Halbert G.W., Pagonis C., Jarman M., Coombes R.C. (1998). Phase I and pharmacokinetic study of D-limonene in patients with advanced cancer. Cancer Research Campaign Phase I/II Clinical Trials Committee. Cancer Chemother. Pharm..

[26] Magrinelli F., Picelli A., Tocco P., Federico A., Roncari L., Smania N., Zanette G., Tamburin S. (2016). Pathophysiology of Motor Dysfunction in Parkinson’s Disease as the Rationale for Drug Treatment and Rehabilitation. Park. Dis..

[27] Nijkrake M.J., Keus S.H., Quist-Anholts G.W., Overeem S., De Roode M.H., Lindeboom R., Mulleners W., Bloem B.R., Munneke M. (2009). Evaluation of a Patient-Specific Index as an outcome measure for physiotherapy in Parkinson’s disease. Eur. J. Phys. Rehabil. Med..

[28] von Wrangel C., Schwabe K., John N., Krauss J.K., Alam M. (2015). The rotenone-induced rat model of Parkinson’s disease: Behavioral and electrophysiological findings. Behav. Brain Res..

[29] Betarbet R., Sherer T.B., MacKenzie G., Garcia-Osuna M., Panov A.V., Greenamyre J.T. (2000). Chronic systemic pesticide exposure reproduces features of Parkinson’s disease. Nat. Neurosci..

[30] Nagatsu T., Nakashima A., Ichinose H., Kobayashi K. (2019). Human tyrosine hydroxylase in Parkinson’s disease and in related disorders. J. Neural Transm (Vienna).

[31] Mohammadi A., Amooeian V.G., Rashidi E. (2018). Dysfunction in Brain-Derived Neurotrophic Factor Signaling Pathway and Susceptibility to Schizophrenia, Parkinson’s and Alzheimer’s Diseases. Curr. Gene.

[32] Wang Y., Liu H., Zhang B.S., Soares J.C., Zhang X.Y. (2016). Low BDNF is associated with cognitive impairments in patients with Parkinson’s disease. Park. Relat. Disord..

[33] Fink A.L. (2006). The aggregation and fibrillation of alpha-synuclein. Acc. Chem. Res..

[34] Sharma L.K., Lu J., Bai Y. (2009). Mitochondrial respiratory complex I: Structure, function and implication in human diseases. Curr. Med. Chem..

[35] Higgins G.C., Beart P.M., Shin Y.S., Chen M.J., Cheung N.S., Nagley P. (2010). Oxidative stress: Emerging mitochondrial and cellular themes and variations in neuronal injury. J. Alzheimers Dis..

[36] Martinez T.N., Greenamyre J.T. (2012). Toxin models of mitochondrial dysfunction in Parkinson’s disease. Antioxid. Redox Signal.

[37] Jenner P., Olanow C.W. (1996). Oxidative stress and the pathogenesis of Parkinson’s disease. Neurology.

[38] Litteljohn D., Mangano E., Clarke M., Bobyn J., Moloney K., Hayley S. (2011). Inflammatory mechanisms of neurodegeneration in toxin-based models of Parkinson’s disease. Park. Dis..

[39] Indo H.P., Yen H.C., Nakanishi I., Matsumoto K., Tamura M., Nagano Y., Matsui H., Gusev O., Cornette R., Okuda T. (2015). A mitochondrial superoxide theory for oxidative stress diseases and aging. J. Clin. Biochem. Nutr..

[40] Roberto D., Micucci P., Sebastian T., Graciela F., Anesini C. (2010). Antioxidant activity of limonene on normal murine lymphocytes: Relation to H2O2 modulation and cell proliferation. Basic Clin. Pharm. Toxicol..

[41] Shah B., Mehta A. (2018). In vitro evaluation of antioxidant activity of D-Limonene. Asian J. Pharm. Pharmacol..

[42] Wang S., Chu C.H., Stewart T., Ginghina C., Wang Y., Nie H., Guo M., Wilson B., Hong J.S., Zhang J. (2015). α-Synuclein, a chemoattractant, directs microglial migration via H2O2-dependent Lyn phosphorylation. Proc. Natl. Acad. Sci. USA.

[43] Le W., Wu J., Tang Y. (2016). Protective Microglia and Their Regulation in Parkinson’s Disease. Front. Mol. Neurosci..

[44] Lorigooini Z., Boroujeni S.N., Sayyadi-Shahraki M., Rahimi-Madiseh M., Bijad E., Amini-Khoei H. (2021). Limonene through Attenuation of Neuroinflammation and Nitrite Level Exerts Antidepressant-Like Effect on Mouse Model of Maternal Separation Stress. Behav. Neurol..

[45] He J., Zhong W., Zhang M., Zhang R., Hu W. (2018). P38 Mitogen-activated Protein Kinase and Parkinson’s Disease. Transl. Neurosci..

[46] Corrêa S.A., Eales K.L. (2012). The Role of p38 MAPK and Its Substrates in Neuronal Plasticity and Neurodegenerative Disease. J. Signal Transduct..

[47] Karunakaran S., Ravindranath V. (2009). Activation of p38 MAPK in the substantia nigra leads to nuclear translocation of NF-κB in MPTP-treated mice: Implication in Parkinson’s disease. J. Neurochem..

[48] Tak P.P., Firestein G.S. (2001). NF-κB: A key role in inflammatory diseases. J. Clin. Investig..

[49] Erekat N.S., Stoker T.B., Greenland J.C. (2018). Apoptosis and its Role in Parkinson’s Disease. Parkinson’s Disease: Pathogenesis and Clinical Aspects.

[50] Zhou Q., Liu C., Liu W., Zhang H., Zhang R., Liu J., Zhang J., Xu C., Liu L., Huang S. (2015). Rotenone induction of hydrogen peroxide inhibits mTOR-mediated S6K1 and 4E-BP1/eIF4E pathways, leading to neuronal apoptosis. Toxicol. Sci..

[51] Choi K.C., Kim S.H., Ha J.Y., Kim S.T., Son J.H. (2010). A novel mTOR activating protein protects dopamine neurons against oxidative stress by repressing autophagy related cell death. J. Neurochem..

[52] Mercado G., Castillo V., Soto P., Sidhu A. (2016). ER stress and Parkinson’s disease: Pathological inputs that converge into the secretory pathway. Brain Res..

[53] Ahn E.H., Kang S.S., Qi Q., Liu X., Ye K. (2020). Netrin1 deficiency activates MST1 via UNC5B receptor, promoting dopaminergic apoptosis in Parkinson’s disease. Proc. Natl. Acad. Sci. USA.

[54] Sahu M.R., Mondal A.C. (2020). The emerging role of Hippo signaling in neurodegeneration. J. Neurosci. Res..

[55] Silva R.M., Ries V., Oo T.F., Yarygina O., Jackson-Lewis V., Ryu E.J., Lu P.D., Marciniak S.J., Ron D., Przedborski S. (2005). CHOP/GADD153 is a mediator of apoptotic death in substantia nigra dopamine neurons in an in vivo neurotoxin model of parkinsonism. J. Neurochem..

[56] Liu W., Wu J., Xiao L., Bai Y., Qu A., Zheng Z., Yuan Z. (2012). Regulation of neuronal cell death by c-Abl-Hippo/MST2 signaling pathway. PLoS ONE.

[57] de Souza M.C., Vieira A.J., Beserra F.P., Pellizzon C.H., Nóbrega R.H., Rozza A.L. (2019). Gastroprotective effect of limonene in rats: Influence on oxidative stress, inflammation and gene expression. Phytomedicine.

[58] Yu L., Yan J., Sun Z. (2017). D-limonene exhibits anti-inflammatory and antioxidant properties in an ulcerative colitis rat model via regulation of iNOS, COX-2, PGE2 and ERK signaling pathways. Mol. Med. Rep..

[59] Chi G., Wei M., Xie X., Soromou L.W., Liu F., Zhao S. (2013). Suppression of MAPK and NF-κB pathways by limonene contributes to attenuation of lipopolysaccharide-induced inflammatory responses in acute lung injury. Inflammation.

[60] Younis N.S. (2020). D-Limonene mitigate myocardial injury in rats through MAPK/ERK/NF-κB pathway inhibition. Korean J. Physiol. Pharmacol..

[61] Pereira E.W.M., Heimfarth L., Santos T.K., Passos F.R.S., Siqueira-Lima P., Scotti L., Scotti M.T., Almeida J., Campos A.R., Coutinho H.D.M. (2022). Limonene, a citrus monoterpene, non-complexed and complexed with hydroxypropyl-β-cyclodextrin attenuates acute and chronic orofacial nociception in rodents: Evidence for involvement of the PKA and PKC pathway. Phytomedicine.

[62] Bacanlı M., Anlar H.G., Aydın S., Çal T., Arı N., Ündeğer Bucurgat Ü., Başaran A.A., Başaran N. (2017). d-limonene ameliorates diabetes and its complications in streptozotocin-induced diabetic rats. Food Chem. Toxicol..

[63] Zhou W., Yoshioka M., Yokogoshi H. (2009). Sub-Chronic Effects of <i>s</i>-Limonene on Brain Neurotransmitter Levels and Behavior of Rats. J. Nutr. Sci. Vitaminol..

[64] Urbach Y.K., Bode F.J., Nguyen H.P., Riess O., von Hörsten S. (2010). Neurobehavioral tests in rat models of degenerative brain diseases. Methods Mol. Biol..

